# 外周血幼淋巴细胞比例在慢性淋巴细胞白血病中的预后价值研究

**DOI:** 10.3760/cma.j.cn121090-20241205-00537

**Published:** 2025-02

**Authors:** 昭亮 张, 家豪 周, 凌霄 邢, 琰 王, 彤璐 邱, 蓉 王, 慧 王, 磊 范, 华渊 朱, 祎 缪, 建勇 李

**Affiliations:** 1 南京医科大学第一附属医院，江苏省人民医院血液科，南京 210029 Department of Hematology, the First Affiliated Hospital of Nanjing Medical University, Jiangsu Province Hospital, Nanjing 210029, China; 2 南京医科大学第一附属医院，江苏省人民医院淋巴瘤中心，南京 210029 Lymphoma Center, The First Affiliated Hospital of Nanjing Medical University, Jiangsu Province Hospital, Nanjing 210029, China

**Keywords:** 白血病，淋巴细胞，慢性，B细胞, 幼淋巴细胞, 布鲁顿酪氨酸激酶抑制剂, Leukemia, lymphocytic, chronic, B-cell, Prolymphocytes, Bruton tyrosine kinase inhibitors

## Abstract

**目的:**

探讨幼淋巴细胞比例对慢性淋巴细胞白血病（CLL）患者预后的影响。

**方法:**

研究纳入了2011年10月至2020年12月在南京医科大学第一附属医院确诊为CLL的300例患者，分析外周血幼淋巴细胞比例与其他基线特征的相关性，然后通过X-tile分析得到外周血幼淋巴细胞比例的最佳临界值，并通过后续的生存分析和预后模型构建验证其预后价值。

**结果:**

共300例符合条件的CLL患者纳入研究，其中有50例CLL患者接受布鲁顿酪氨酸激酶抑制剂（BTKi）作为一线治疗。外周血幼淋巴细胞比例较高的患者多为晚期（*P*＝0.010）、β_2_微球蛋白较高（*P*<0.001）、免疫球蛋白重链可变区（IGHV）未突变（*P*<0.001）和伴有TP53异常（*P*＝0.004）。外周血幼淋巴细胞比例的最佳临界值为1％。外周血幼淋巴细胞比例高于1％的患者的无治疗生存（TFS）期（*P*<0.001）和总生存期（*P*＝0.007）短。在多因素分析中，外周血幼淋巴细胞比例>1％对TFS的独立预测价值尚不明确，但表现出一定的趋势［*HR*＝1.405（95％ *CI* 0.971～2.032），*P*＝0.071］。将CLL国际预后指数（CLL-IPI）和外周血幼淋巴细胞比例结合构建的新预后模型比CLL-IPI有更好的区分度［曲线下面积（AUC）：0.778对0.637，*P*＝0.006］。此外，外周血幼淋巴细胞比例>1％的患者在接受布鲁顿酪氨酸激酶抑制剂治疗后无进展生存期更短（*P*＝0.038）。

**结论:**

外周血幼淋巴细胞比例与治疗前CLL患者的基线特征及预后相关。

## Abstract (en)

**Objective:**

To investigate the impact of peripheral blood prolymphocyte percentage on the prognosis of patients with chronic lymphocytic leukemia（CLL）.

**Methods:**

This study included 300 patients diagnosed with CLL at the Department of Hematology of Jiangsu Provincial People's Hospital from October 2011 to December 2020. The association between prolymphocyte percentage and other parameters was analyzed, and the optimal cutoff prolymphocyte percentage was determined by X-tile analysis. Further survival analysis and prognostic model construction were used to validate the predictive value of prolymphocyte percentage.

**Results:**

Of the 300 eligible patients with CLL who were enrolled, 50 received Bruton tyrosine kinase inhibitors（BTKi）as first-line treatment. The group with higher prolymphocyte percentage comprised more patients in the advanced stages（*P*＝0.010）and had higher β_2_-microglobulin（*P*<0.001）, unmutated immunoglobulin heavy-chain variable region gene（*P*<0.001）, and TP53 aberration（*P*＝0.004）. The optimal cutoff percentage of prolymphocytes was 1％. Patients with a prolymphocyte percentage >1％ had significantly shorter treatment-free survival（TFS）（*P*<0.001）and overall survival time（*P*＝0.007）than patients with a prolymphocyte percentage ≤1％. On multivariate analysis, prolymphocyte percentage >1％ tended to have an independent prognostic value for TFS［*HR*＝1.405（95％ *CI* 0.971～2.032），*P*＝0.071］. Compared with the nomogram of CLL international prognostic index（CLL-IPI）alone, the nomogram of CLL-IPI combined with prolymphocyte percentage showed better discrimination（area under the curve: 0.778 *vs*. 0.637; *P*＝0.006）. In addition, patients with a prolymphocyte percentage >1％ were more likely to progress after BTKi treatment（*P*＝0.038）.

**Conclusion:**

Peripheral blood prolymphocyte percentage was associated with various clinical and biological parameters and prognosis among patients with treatment-naive CLL.

慢性淋巴细胞白血病（CLL）是一种成熟B淋巴细胞增殖性疾病，以外周血、骨髓或淋巴组织中出现CD5^+^CD23^+^小淋巴细胞为特征。CLL的病程具有异质性，一些患者在诊断后需立即治疗，病程进展迅速，而另一些患者病程相对惰性，可观察等待数年仍无需治疗[1]。CLL国际预后指数（CLL-IPI）包括临床分期、年龄、β_2_微球蛋白、免疫球蛋白重链可变区（IGHV）突变状态、TP53异常共5个因素，是目前最为普遍应用的CLL预后模型[2]–[3]。外周血涂片是CLL重要的诊断方法，多项研究表明外周血幼淋巴细胞比例越高，CLL患者预后越差，对传统疗法的治疗反应越差[4]–[8]。然而，外周血幼淋巴细胞比例预测CLL预后的最佳截断值仍不明确，且在中国人群中尚无关于其预后意义的相关研究。因此，本研究在单中心队列中探索外周血幼淋巴细胞比例在CLL中的预后价值。

## 病例与方法

1. 病例：回顾性纳入2011年10月至2020年12月在南京医科大学第一附属医院血液科根据国际慢性淋巴细胞白血病研讨会标准（iwCLL）[9]确诊为CLL的初诊患者共410例，其中300例患者有保留的基线外周血涂片可供分析，因此共纳入300例符合条件的患者。300例患者中有50例患者接受布鲁顿酪氨酸激酶抑制剂（BTKi）作为一线治疗。本研究豁免知情同意且经南京医科大学第一附属医院伦理委员会批准（批件号：2021-SRFA-202）。

2. 外周血涂片幼淋巴细胞比例：由形态学专家对用May-Griinwald-Giemsa技术染色的入组患者外周血涂片进行阅片。阅片区域选取涂片主体与尾部的交界处。幼淋巴细胞为染色质呈团块状、核仁呈大的突出泡状、通常有丰富细胞质的大细胞。在血涂片中共计数200个淋巴细胞，并记录幼淋巴细胞百分比。

3. 临床信息统计：基线信息包括年龄、性别、Rai和Binet分期、β_2_微球蛋白、LDH、CD38表达、Zeta链相关蛋白激酶70（ZAP70）表达、HGB、PLT、淋巴细胞绝对计数、白蛋白、ATM状态、12号染色体状态、染色体核型、IGHV突变状态和TP53状态。

4. 随访：通过住院病历及门诊病历系统获取患者基本情况及实验室结果，并通过电话随访进一步调研，随访截止时间为2021年10月30日。无治疗生存（TFS）期指从血涂片采样到首次治疗的时间，无进展生存（PFS）期指从治疗到出现复发或进展的时间，总生存（OS）期指从血涂片采样到死亡或最后一次随访的时间。

5. 统计学处理：计数资料以例数（百分比）表示，组间比较采用卡方检验；计量资料以*M*（范围）表示，组间比较采用Wilcoxon秩和检验。应用X-tile软件判断外周血幼淋巴细胞比例的最佳截断值。采用Kaplan-Meier法绘制生存曲线，并用Log-rank检验比较组间生存差异。采用Cox回归模型进行多因素分析。联合CLL-IPI的5个参数和外周血幼淋巴细胞比例构建预后模型，并用列线图展示。受试者工作特征曲线（ROC）和曲线下面积（AUC）用来评估模型区分度。DeLong检验用于比较AUC之间的差异。对于部分临床信息缺失的数据采用了列表删除的方式，即在分析某一变量时，排除了该变量缺失的病例。使用R软件和Graphpad prism进行统计分析和图表绘制。所有检验均为双侧检验，*P*<0.05为差异有统计学意义。

## 结果

1. 基线特征：300例患者自诊断到外周血涂片检查的中位时间为0.2（0～108）个月，中位随访时间为40.5（0.1～156.1）个月。中位TFS期为14.4（95％ *CI* 5.0～23.8）个月，中位OS期未达到。如表1所示，300例患者的中位年龄为62（29～85）岁，其中38.7％（116/300）的患者年龄在65岁以上。87％（261/300）患者为晚期（RaiⅠ～Ⅳ期或Binet B～C期）。38％（117/300）和22％（66/300）的患者出现β_2_微球蛋白和LDH升高。39.3％（96/244）患者的IGHV突变状态为未突变，14％（31/221）的患者存在TP53异常，包括TP53突变和（或）缺失。外周血幼淋巴细胞比例中位数为0（0～40）％。

**表1:** 300例初诊慢性淋巴细胞白血病患者的基线特征

基线特征	例（％）
年龄>65岁	116（38.7）
男性	202（67.3）
RaiⅠ～Ⅳ期或Binet B～C期	261（87.0）
β_2_微球蛋白>3.5 mg/L	113（37.7）
LDH>正常值上限	66（22.0）
HGB<100 g/L	52（17.3）
PLT<100×10^9^/L	74（24.7）
淋巴细胞绝对计数>30×10^9^/L	123（41.0）
白蛋白<30 g/L	14（4.7）
CD38≥30％^a^	33（22.8）
ZAP70≥20％^a^	37（39.4）
ATM缺失^a^	18（7.8）
13q−^a^	99（46.5）
+12^a^	42（19.8）
复杂核型^a^	30（15.9）
IGHV未突变^a^	96（39.3）
TP53异常^a^	31（14.0）
TP53突变^a^	27（11.5）
TP53缺失^a^	23（8.7）

**注** ^a^该项部分患者数据缺失，计算时已将其从总数中删去；IGHV：免疫球蛋白重链可变区

2. 外周血幼淋巴细胞比例同其他基线特征的相关性：Rai或Binet分期晚期（*P*＝0.010），β_2_微球蛋白（*P*<0.001）、LDH（*P*<0.001）、淋巴细胞绝对计数（*P*＝0.027）和CD38表达较高（*P*<0.001），HGB较低（*P*<0.001），无13q−（*P*＝0.009），复杂核型（*P*＝0.016），IGHV无突变（*P*<0.001）和TP53异常（*P*＝0.004）的患者外周血幼淋巴细胞比例较高。

3. 明确外周血幼淋巴细胞比例的最佳截断值及预后价值：利用X-tile软件得到预测TFS和OS的外周血幼淋巴细胞比例的最佳截断值均为1％。外周血幼淋巴细胞比例>1％的患者TFS期和OS期短于≤1％的患者［中位TFS期：1.4（95％ *CI* 0～3.2）个月对41.7（95％ *CI* 9.2～74.2）个月，*P*<0.001；中位OS期均未达到，*P*＝0.007］（图1）。Oscier等[10]的研究将5％和10％作为外周血幼淋巴细胞比例的截断值，因此我们也验证了这两个截断值在预测TFS和OS方面的作用。外周血幼淋巴细胞比例>5％或10％，TFS期均较短（均*P*<0.05）（图2A、B），但OS差异均无统计学意义（均*P*>0.05）（图2C、D）。

**图1:**
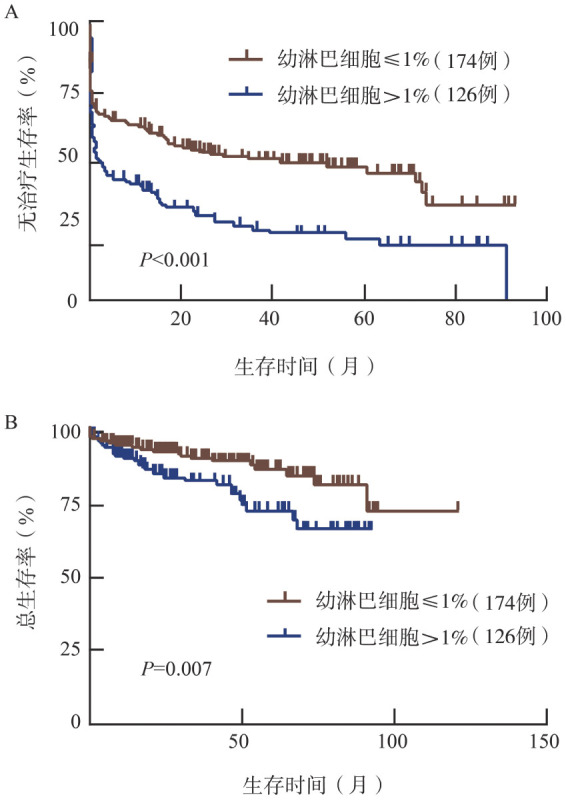
以1％作为外周血幼淋巴细胞比例截断值的慢性淋巴细胞白血病患者的无治疗生存曲线（A）和总生存曲线（B）

**图2:**
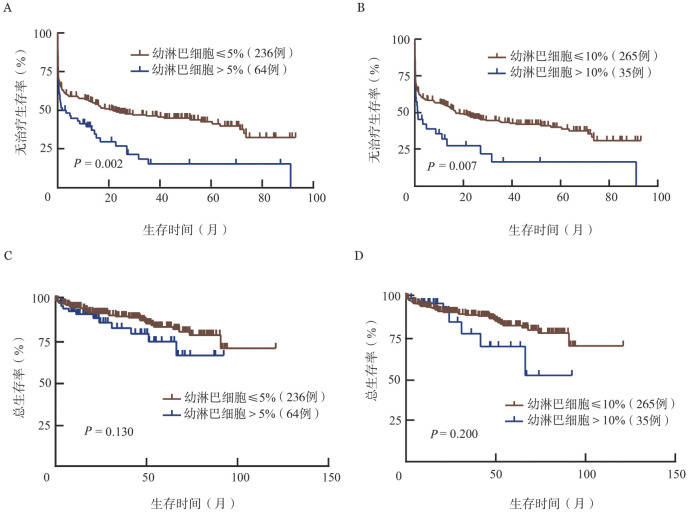
以5％或10％作为外周血幼淋巴细胞比例截断值的慢性淋巴细胞白血病患者的无治疗生存曲线（A、B）和总生存曲线（C、D）

进一步将年龄、Rai或Binet分期、β_2_微球蛋白、IGHV突变状态、TP53状态和幼淋巴细胞百分比纳入多因素分析，发现外周血幼淋巴细胞比例>1％对OS没有独立的预后价值［*HR*＝1.051（95％ *CI* 0.433～2.550），*P*＝0.913］，对TFS的独立预测价值尚不明确，但表现出一定的趋势［*HR*＝1.405（95％ *CI* 0.971～2.032），*P*＝0.071］（表2）。

**表2:** 多因素分析外周血幼淋巴细胞比例的预后价值

变量	无治疗生存		总生存	
	*HR*（95％ *CI*）	*P*值	*HR*（95％ *CI*）	*P*值
幼淋巴细胞比例>1％	1.405（0.971～2.032）	0.071	1.051（0.433～2.550）	0.913
年龄>65岁	0.604（0.411～0.889）	0.011	2.914（1.219～6.965）	0.016
β_2_微球蛋白>3.5 mg/L	1.947（1.335～2.839）	0.001	2.453（0.911～6.605）	0.076
Rai Ⅰ～Ⅳ期或Binet B～C期	2.654（1.063～6.629）	0.037	0.510（0.101～2.583）	0.416
IGHV无突变	1.478（1.022～2.137）	0.038	2.127（0.806～5.613）	0.128
TP53异常	1.414（0.873～2.290）	0.160	1.578（0.558～4.462）	0.389

**注** IGHV：免疫球蛋白重链可变区

4. 不同外周血幼淋巴细胞比例的患者在临床或实验室指标方面的差异：为了进一步研究外周血幼淋巴比例的临床和生物学意义，我们将所有患者分为3组，幼淋巴细胞比例≤1％（A组，174例患者）、1％<幼淋巴细胞比例≤5％（B组，62例患者）和幼淋巴细胞比例>5％（C组，64例患者）。幼淋巴细胞比例>1％为BC组。如表3所示，与A组相比，BC组患者有较晚的分期，较高的β_2_微球蛋白、CD38表达、LDH、淋巴细胞绝对计数以及较低的HGB和PLT，且BC组IGHV无突变和TP53异常的患者比例更高。以5％作为截断值的B组和C组在分期、HG、PLT数、CD38表达及IGHV突变状态和TP53状态方面差异均无统计学意义。以上研究结果为将1％作为最佳截断值提供了依据。

**表3:** 分别以1％和5％作为外周血幼淋巴细胞比例截断值的慢性淋巴细胞白血病患者基线特征的组间差异比较［例数（％）］

基线特征	A（174例）	BC（126例）	B（62例）	C（64例）	A对BC		A对B		B对C	
					*χ*^2^值	*P*值	*χ*^2^值	*P*值	*χ*^2^值	*P*值
年龄>65岁	63（36.2）	53（42.1）	29（46.8）	24（37.5）	1.057	0.304	2.146	0.143	1.111	0.292
男性	112（64.4）	90（71.4）	43（69.4）	47（73.4）	1.656	0.198	0.507	0.478	0.257	0.612
Rai I～IV或Binet B～C	144（82.8）	117（92.9）	56（90.3）	61（95.3）	6.589	0.010	2.023	0.155	1.182	0.277
β_2_微球蛋白>3.5 mg/L	43（24.7）	70（55.6）	27（43.5）	43（67.2）	29.610	<0.001	7.773	0.005	7.127	0.008
LDH>正常值上限	24（13.8）	42（33.3）	14（22.6）	28（43.8）	16.261	<0.001	2.613	0.106	6.351	0.012
HGB<100 g/L	17（9.8）	35（27.8）	18（29.0）	17（26.6）	16.539	<0.001	13.428	<0.001	0.096	0.757
PLT<100×10^9^/L	35（20.1）	39（31.0）	22（35.5）	17（26.6）	4.619	0.032	5.894	0.015	1.173	0.279
绝对淋巴细胞计数>30×10^9^/L	63（36.2）	60（47.6）	28（45.2）	32（50.0）	3.935	0.047	1.547	0.214	0.296	0.587
白蛋白<30 g/L	6（3.4）	8（6.3）	3（4.8）	5（7.8）	1.382	0.240	0.241	0.624	0.468	0.494
CD38≥30％^a^	11（13.1）	22（36.1）	9（29.0）	13（43.3）	10.607	0.001	4.003	0.045	1.352	0.245
ZAP70≥20％^a^	19（37.3）	18（41.9）	9（37.5）	9（47.4）	0.207	0.649	0.000	0.984	0.424	0.515
ATM缺失^a^	9（6.9）	9（8.9）	3（6.1）	6（11.5）	0.313	0.565	0.036	0.858	0.912	0.340
13q−^a^	66（55.0）	33（35.5）	19（43.2）	14（28.6）	8.022	0.005	1.801	0.180	2.162	0.141
+12^a^	14（11.7）	28（30.4）	7（15.9）	21（43.8）	11.546	0.001	0.519	0.471	8.404	0.004
复杂核型^a^	17（15.6）	13（16.5）	3（8.3）	10（23.3）	0.025	0.874	1.201	0.273	3.174	0.075
IGHV未突变^a^	39（27.9）	57（54.8）	24（47.1）	33（62.3）	18.162	<0.001	6.235	0.013	2.426	0.119
TP53异常^a^	10（8.3）	21（21.0）	9（20.0）	12（21.8）	7.364	0.007	4.457	0.035	0.490	0.824
TP53突变^a^	10（7.6）	17（16.5）	6（13.0）	11（19.3）	4.536	0.033	1.247	0.264	0.723	0.395
TP53缺失^a^	6（4.1）	17（14.5）	6（10.3）	11（18.6）	8.942	0.003	2.960	0.085	1.622	0.203

**注** IGHV：免疫球蛋白重链可变区；^a^该项部分患者数据缺失，计算时已将其从总数中删去

5. 构建新的预后模型并评估其区分度：我们联合CLL-IPI和外周血幼淋巴细胞比例构建新的预后模型，以列线图的形式展示，分别预测未经治疗的CLL患者的TFS（图3A）和OS（图3B）。预后模型预测TFS和OS的AUC分别为0.778和0.737。与CLL-IPI相比，新预后模型显著提高了TFS的区分度（0.778对0.637，*P*＝0.006），但对OS的区分度提高不明显（0.737对0.712，*P*＝0.717）（图3C、D）。

**图3:**
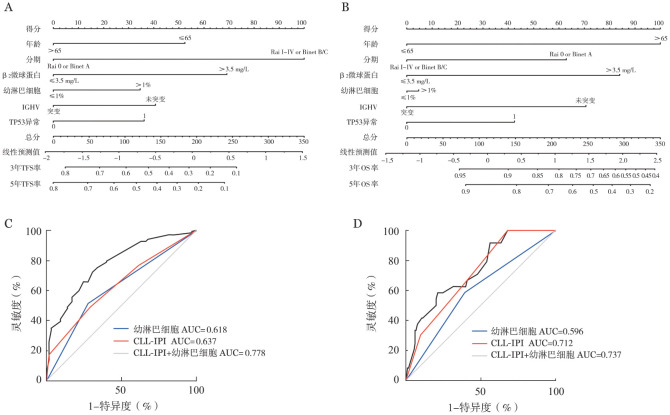
预测无治疗生存（TFS）及总生存（OS）的列线图（A、B）及受试者工作特征曲线（C、D） **注** IGHV：免疫球蛋白重链可变区；AUC：曲线下面积

6. 外周血幼淋巴细胞比例对接受布鲁顿酪氨酸激酶抑制剂（BTKi）作为一线治疗的CLL患者PFS的影响：我们进一步分析了50例接受BTKi单药或联合疗法作为一线治疗的患者。18例（36％）患者接受了BTKi单药治疗，其中16例接受了伊布替尼治疗、2例接受了泽布替尼治疗。其余患者（32/50，64％）接受了联合疗法：18例接受伊布替尼/泽布替尼联合FCR（氟达拉滨+环磷酰胺+利妥昔单抗）方案，其他组合方案包括BR（苯达莫司汀+利妥昔单抗）方案、利妥昔单抗和VR（维奈克拉+利妥昔单抗）方案。外周血幼淋巴细胞比例>1％的患者（27/50，54％）在接受BTKi治疗后更短时间出现疾病进展（*P*＝0.038）（图4）。两组患者的中位PFS期均未达到。

**图4:**
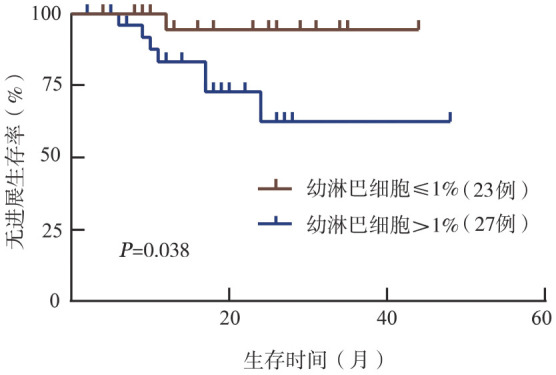
以1％的外周血幼淋巴细胞比例分组的慢性淋巴细胞白血病患者接受布鲁顿酪氨酸激酶抑制剂治疗后的无进展生存曲线

## 讨论

CLL细胞的形态反映了其生物学特征。幼淋巴细胞增多表明细胞分裂增加，蛋白质生成增强，这也是临床病程更具侵袭性、TFS期更短的原因。幼淋巴细胞增加与高风险分子或遗传学特征的存在有关，包括未突变的IGHV和TP53异常，这与之前的研究一致[11]–[12]。此外，我们还发现CLL-IPI中不包括的复杂核型也与外周血幼淋巴细胞比例增加有关。复杂核型已被证明是接受化疗、免疫疗法或BTKi治疗的CLL患者的不良预后因素[13]–[14]。

已有多项研究探讨幼淋巴细胞与生存的关系，然而不同研究的结论并不一致，幼淋巴细胞的截断值也未得到证实。Dubner等[5]发现，淋巴细胞体积较大且含有5％以上带核仁淋巴细胞的CLL患者生存率较低。Economopoulos等[6]的研究表明，幼淋巴细胞比例超过10％的患者更有可能表现为晚期、高淋巴细胞计数且更容易进展为难治性。B细胞幼淋巴细胞白血病的诊断标准是外周血幼淋巴细胞占淋巴细胞的比例超过55％，而典型CLL的幼淋巴细胞比例应少于10％[15]。幼淋巴细胞比例为11％～55％的患者被称为幼淋巴细胞增多的CLL，其生存率低于典型CLL。对于这部分患者，幼淋巴细胞绝对计数≥15×10^9^/L预示着生存较差[16]。Vallespí等[8]进一步指出，较高的幼淋巴细胞比例（>5％或>10％）和幼淋巴细胞绝对计数（>5×10^9^/L或>15×10^9^/L）可区分不同的风险组别。Oscier等[17]的研究也证实，与典型CLL相比，这部分患者的PFS期更短。幼淋巴细胞百分比越高，预后越差，但上述研究中使用5％或10％作为幼淋巴细胞比例的截断值并不具有充分的依据。本研究通过使用生物信息学工具X-tile将1％定义为预测患者预后的外周血幼淋巴细胞比例的最佳截断值。外周血幼淋巴细胞比例>1％的患者TFS期和OS期都较短。本研究证实外周血幼淋巴细胞比例为1％～5％的CLL患者，其未突变的IGHV和TP53异常的发生率高于外周血幼淋巴细胞≤1％的患者，但与外周血幼淋巴细胞比例>5％的患者相似。表明外周血幼淋巴细胞比例为1％～5％的CLL更接近幼淋巴细胞比例>5％的CLL，进一步支持将1％作为最佳截断值。

我们认为在预测CLL患者的预后时，外周血幼淋巴细胞比例的最佳截断值是1％，而不是5％或10％。这一结果与部分国外研究结论有所差异，可能与患者群体的遗传背景、疾病分型及研究方法学不同有关。例如，亚洲CLL患者中IGHV基因未突变的比例较低[18]，而这一亚型患者往往伴有较高的幼淋巴细胞比例。其次，我们选择了TFS作为终点，这与其他研究不同，其受治疗方案影响的可能性较小。此外，本研究采用的X-tile方法具有更高的统计学严谨性，能够从生物学意义上识别最佳预测值。进一步的分组研究表明，幼淋巴细胞比例≤1％和1％～5％的患者在经典预后标志物方面有显著差异。Matutes等[19]提出12号染色体三体定义了一个非典型形态更常见的亚组。本队列中，这部分患者的外周血幼淋巴细胞比例往往较高。虽然在多因素分析中，外周血幼淋巴细胞比例对预后的影响只有趋势，但比例越高，TFS期越短，将外周血幼淋巴细胞比例和CLL-IPI结合起来，可以更准确地预测治疗前CLL患者的TFS。本研究队列中，65岁以上患者的TFS优于年轻患者。我们推测，这可能是由于中国老年患者的定期随访意识不强。BTKi在未经治疗的CLL患者中效果显著，改变了CLL的治疗格局[20]–[22]。本研究外周血幼淋巴细胞比例>1％的患者疾病进展时间更短。这表明外周血幼淋巴细胞比例在预测BTKi疗效方面有一定作用。

本研究存在局限性。在没有免疫分型特征的情况下仅依靠形态学特征识别幼淋巴细胞较为主观。研究数据未包括免疫表型、SmIg和FMC7表达、淋巴结和脾脏侵犯情况等因素，这些因素在既往报道中与幼淋巴细胞有关[16],[23]。此外，本研究基于单中心数据，可能存在一定的地域和就诊人群选择偏倚，因此本研究中的患者无法完全代表中国CLL患者的总体情况，需要通过多中心和全国范围的大样本研究进一步验证结论的普适性。由于部分患者数据的缺失，可能会限制研究结果的广泛性和外推性，未来研究中，需进一步完善数据收集工作。样本量小（仅限于中国患者）以及随访时间短也是明显的局限。

总之，本研究提示，外周血幼淋巴细胞比例越高，预后越差，并首次报道了外周血幼淋巴细胞比例与较短的TFS期相关。尽管在多变量分析中未显示独立的预后作用，但1％作为外周血幼淋巴细胞比例截断值仍能区分不同的预后。结合CLL-IPI和外周血幼淋巴细胞比例的新预后模型被证明具有更好的预测准确性。研究进一步分析显示外周血幼淋巴细胞比例较高的患者在接受BTKi或基于BTKi的治疗方案后，PFS较差。因此，在未来的临床工作中关注幼淋巴细胞，有助于CLL诊疗工作更好地进行。
